# Phenotypic plasticity of European larch radial growth and wood density along a‐1,000 m elevational gradient

**DOI:** 10.1002/pei3.10040

**Published:** 2021-02-20

**Authors:** Margarita Escobar‐Sandoval, Luc Pâques, Patrick Fonti, Alejandro Martinez‐Meier, Philippe Rozenberg

**Affiliations:** ^1^ INRAE UMR 0588 BIOFORA Orléans Cedex 2 France; ^2^ Swiss Federal Institute for Forest Snow and Landscape Research WSL Birmensdorf Switzerland; ^3^ INTA EEA Bariloche Grupo de Ecología Forestal San Carlos de Bariloche, Río Negro Argentina

**Keywords:** Adaptation, Cline, dendroecology, *Larix decidua*, microdensity, plastic, reaction norm, response curve, temperature, tree‐ring

## Abstract

Phenotypic plasticity is a key mechanism for sedentary long‐living species to adjust to changing environment. Here, we use mature *Larix decidua* tree‐ring variables collected along an elevational transect in the French Alps to characterize the range of individual plastic responses to temperature. Stem cores from 821 mature *Larix decidua* trees have been collected from four plots distributed along a 1,000‐m elevational gradient in a natural forest to build up individual linear reaction norms of tree‐ring microdensity traits to temperature. The sign, magnitude and spread of variations of the slopes of the individual reaction norms were used to characterize variation of phenotypic plasticity among plots and traits. Results showed a large range of phenotypic plasticity (with positive and negative slopes) at each elevational plot and for each tree‐ring variable. Overall, phenotypic plasticity tends to be larger but positive at higher elevation, negative at the warmer lower sites, and more variable in the center of the elevation distribution. Individual inter‐ring reaction norm is a valuable tool to retrospectively characterize phenotypic plasticity of mature forest trees. This approach applied to *Larix decidua* tree‐ring micro‐density traits along an elevation gradient showed the existence of large inter‐individual variations that could support local adaptation to a fast‐changing climate.

## Graphical abstract

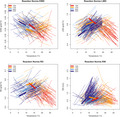

## 1 INTRODUCTION

Phenotypic Plasticity (abbreviated as PP in the article) commonly refers to the ability of a genotype (an individual's gene collection) to adjust its phenotype (the set of observable traits of the same individual) under different environmental conditions (Arnold et al., 2019; Bradshaw, 1965; Pigliucci & Pigliucci, 2001). This mechanism plays a crucial role for the development and survival of individuals facing new adverse environmental conditions (Silvestre et al., 2012), especially if we refer to sedentary and long‐living organism such as forest trees (Bradshaw, 1965; Santini et al., 2018). Due to their long existence, trees have to face climatic fluctuations of variable frequencies and intensities (Rehfeldt et al., 2001) and thus have higher chances to come across extreme weather events. PP however is also a very important mechanism to adapt to rapid environmental change (Bradshaw & Holzapfel, 2008): PP is the only possible adaptation mechanism if the length of the change is much less than the generation time (Bradshaw, 1965). However, despite the accelerated interest in unravelling the genetic basis of PP in relation to the fast‐increasing global warming, its quantification for the endangered sedentary and long‐living organisms remains a challenge and is scarce.

PP is usually estimated using Reaction Norm (RN), which is a linear or non‐linear function indicating the amount of phenotypic change of a genotype between environments (Arnold et al., 2019; Falconer, 1990; Morrissey & Liefting, 2016; Via et al., 1995). Generally, the environmental variability is generated by exposing the same genotype to different conditions. Spatial (site‐related) and temporal (time‐related) PP refer to the types of environmental variation (Scheiner, 2013). The spatial PP requires reciprocal experiments and copies of the same genotype in different sites or controlled conditions (Via, 1993; Vitasse et al., 2010). The temporal PP demands repeated measurements (Araya‐Ajoy et al., 2015) or retrospective evaluations (Fonti et al., 2010; Marchal et al., 2019). While the definition is simple, accurate spatial (or site‐related) RN are often difficult to estimate, since the number of sites or controlled conditions is generally low and their environmental value is most of the time problematic to quantify (Pigliucci & Pigliucci, 2001). Temporal (or time‐related) PP can be estimated from series of repeated measurements, or, for some organisms like fish and tree species, retrospectively using temporal markers (scales in fish and annual rings in trees; Black et al., 2019; Fallour‐Rubio et al., 2009). Estimation of temporal PP is never straightforward, since it can be confused with the ontogenic development of the organism.

Trees have a high potential for recording signals at different temporal scales and frequencies. Many studies explored tree response to environmental variation based on radial growth, annual ring formation and relationships with climate estimated at the population level. In particular, dendrochronology (Büntgen, 2019; Vitas, 2018) and dendroecology (Manzanedo & Pederson, 2019; Pompa‐García et al., 2018; Vitasse et al., 2019) achieve climatic reconstructions and ecological investigations (Huang et al., 2017) through retrospective measurements of ring width or, more recently, basal area increments (Biondi & Qeadan, 2008). Typically, dendroecology estimates and employs a response curve that corresponds to a RN calculated at the level of the group of individuals (population): it thus estimates the average plasticity of this group of individuals, and it can be called *average plastic response* (Feinard‐Duranceau et al., 2018). Other physical and anatomical ring variables are rarely used as alternatives to ring width (Fonti & Jansen, 2012). Non‐significant average plastic response may conceal very variable levels of PP, which can be elucidated only by fitting RN at the individual level, and fails to achieve the key‐question about the variability and significant differences between entities (groups of trees, populations, genetic entities) for PP (Arnold et al., 2019). But developing significant^1^ RN, that is, significant phenotype‐environment relationships at the genotype, that is, at the individual level, is challenging and rarely achieved in PP studies (Arnold et al., 2019). This may explain why, while the amount of dendroecology studies estimating an average plastic response is very high, and while, to our knowledge, the first published study of inter‐ring PP is relatively old (Fallour‐Rubio et al., 2009), the number of published studies estimating PP by means of individual inter‐ring RN is still very low today.

The retrospective use of tree‐rings for analysis of PP with construction of RN involves two different time‐scale approaches: *inter*‐ and *intra*‐ring PP. The inter‐ring approach considers inter‐annual PP, while the intra‐ring methodology investigates intra‐annual (or intra‐growing season) PP. Studies of inter‐ring PP associate inter‐annual time‐series of ring variables to inter‐annual time‐series of climatic variables, like for example ring width and mean summer temperature (Fallour‐Rubio et al., 2009) or ring width and a climatic drought index (Marchal et al., 2019). Intra‐ring PP considers the construction of a RN associating intra‐ring time‐series with intra‐annual environmental variables, such as ring microdensity profiles and water balance variation during the year (Sánchez‐Vargas et al., 2007). Intra‐annual ring fluctuations (IADF) are also used as a way to quantify intra‐ring PP (Balzano et al., 2019; Nabais et al., 2014).

The shape of the RN, linear or non‐linear, determines the variables used for eventually estimating the PP. If the RN is linear, the slope of the straight regression line is the unique PP variable (Arnold et al., 2019; Sánchez‐Vargas et al., 2007). If the RN is non‐linear, then several variables may be necessary to correctly quantify the PP (Martinez‐Meier et al., 2009). These authors proposed the word “dendroplasticity” to describe the retrospective estimation of PP based on tree‐ring analysis.

The slope of the RN, like any other phenotypic trait, can be genetically (Fallour‐Rubio et al., 2009; Martinez‐Meier et al., 2009; Sánchez‐Vargas et al., 2007) and environmentally (Marchal et al., 2019) variable. (Martinez‐Meier et al., 2009; Sánchez‐Vargas et al., 2007) found significant genetic determinism for PP of intra‐ring microdensity in response to climatic indices.

In this study, we use microdensity tree‐ring variables of European Larch (*Larix decidua* Mill.) to estimate inter‐annual PP to temperature, aiming at maximizing the number of trees with significant RN estimation at all elevation levels and for all ring variables. Then we study PP variation between individuals and between contrasted environments, that is, four study plots along an elevational gradient in a naturally regenerated adult Larch forest. We selected microdensity variables considering that density is a proxy for important xylem ecological (Björklund et al., 2019) and functional properties (e.g. hydraulic efficiency and safety and mechanical stability) (Lachenbruch & McCulloh, 2014).

European larch is a key forest tree species all over the Alps and in certain regions of Central Europe. In the southwestern part of its natural area, Larch is distributed along elevation gradients starting as low as 1,200 m and culminating as high as 2,500 m. It is one of the mountain species with the major temperature variation along its elevational gradients (Carrer et al., 1998; Fourchy, 1952; Jochner et al., 2017; Obojes et al., 2018; Saulnier et al., 2019). Elevational gradients are the most powerful natural experiments to test ecological responses to geophysical influences, such as temperature (Körner, 2007). Individuals in colder conditions increase their growth when temperature increases, while on the contrary individuals in warmer condition reduce their growth when temperature increases, with a smaller between‐individual variation at the warmer site (Clark et al., 2003; Way & Oren, 2010). This suggests that their response to time‐related temperature variation and specifically to global warming is distinct at the different temperature conditions of the various elevational levels (Morin et al., 2018). Populations distributed over an environmental gradient experience differential selection pressures (Barton, 1999), which can ultimately lead to different levels of intra‐population variation and inter‐population genetic differentiation for adaptive traits. Local adaptation occurs when populations that experience divergent selection become better adapted to their own local environment than other populations, and comparatively less adapted to neighboring ones (Kawecki & Ebert, 2004). The other possibility is that the larch forest distributed along this gradient be one predominantly homogeneous population with considerable gene flow between all elevational levels (King et al., 2013). In fact this pattern was observed with the same trees used in this study along the same elevational gradient for neutral genetic markers (Nardin et al., 2015). Whether variation of larch PP reflects local adaptation or neutral genetic variation is among the questions raised by this study.

Our objective was to fit inter annual‐ring reaction norms of several ring variables and use their variations in sign, magnitude and range to estimate PP variation among elevational plots and ring variables. Finally, we discuss whether elevational variation for PP could correspond to local adaptation and could improve larch response to climate change.

## 2 MATERIAL AND METHODS

### 2.1 Sites characteristics

Our study site is located in the native range of *Larix decidua* (Mill.) at Villard‐St‐Pancrace, close to Briançon (latitude: 44.9° N; longitude: 6.65° E; average elevation: 1,326 m) in the French Alps. The experiment is formed of four forest plots distributed along a north‐faced Alpine steep‐slope ranging from 1,200 m to 2,500 m and mostly covered by uneven‐aged larch forests with some patches of *Abies alba*, *Pinus sylvestris*, *Pinus cembra* and *Pinus uncinata*. The four plots each include 200 larch trees and are located at 2,300, 2,000, 1,700 and 1,350 m a.s.l., respectively (Nardin, 2013; Nardin et al., 2015). The average annual maximum temperature difference along the gradient is 6.1°C, changing from 7.58°C at the highest site to 13.68°C at the lowest one (Table 1; Table S1). Besides temperature, other factors such as solar radiation (higher at the lowest plots), and soil water availability and fertility (higher for the two intermediate plots) are changing. These differing conditions affect the tree characteristics at each plot with large variation on average tree height (from 16 to 27 m), stem girth (from 81 to 112 cm) and average annual radial growth (from 0.63 to 0.91 mm; Table 1; Table S1).

**TABLE 1:** Site and plots characteristics. Values in parenthesis are standard errors. The climatic data are for the period 1967–2007 (source Météo‐France)

	Plot 2,300 m	Plot 2,000 m	Plot 1,700 m	Plot 1,350 m
Altitude range (m)	2,357–2,299	2,023–1,988	1,683–1,640	1,373–1,341
Latitude (N)	44.848972	44.85119	44.858476	44.854350
Longitude (E)	6.636990	6.628716	6.624955	6.597923
Mean annual maximum temperature (°C)	7.58	9.56	11.96	13.68
Mean annual temperature (°C)	2.92	4.46	6.32	7.66
Mean temperature of the warmest month; July (°C)	10.95	12.95	15.37	17.09
Mean temperature of the coldest month; January (°C)	−3.73	−2.72	−1.49	−0.61
Average number of frost days	208	192	170	154
Soil type	Calcisol	Eutric Brunisol / Calcisol	Colluviosol	Regosol / Colluviosol
Total number of trees in the plot	198	217	206	200
Plot size (m^2^)	5,429	7,540	5,815	8,704
Plot density (nb trees/ha)	365	288	354	230
Mean height (m)	16.21 (± 2.19)	25.31 (± 2.76)	26.71 (± 2.93)	23.79 (± 2.64)
Mean circumference at breast height (cm)	79.3 (± 27.4)	98.6 (± 32.8)	105.1 (± 34.4)	107 (± 25.4)
Average ring number in the increment cores, as an estimation of tree age	95.7 (± 34.1)	134.0 (± 48.8)	133.2 (± 46.1)	143.7 (± 15.5)

### 2.2 X‐ray, cross‐dating and detrending

All the details about the methods of tree‐ring related measurements are in (Nardin, 2013; Nardin et al., 2015; Rozenberg, Chauvin, et al., 2020; Rozenberg et al., 2020). Shortly, from each selected tree, we collected a 5.5 mm diameter‐increment core at 1.3 m in the stem using a power‐driven Pressler increment borer. The increment cores were stored in polycarbonate honeycomb boxes and dried to reach a stable uniform humidity of about 12%. The cores were sawn to a uniform cross‐section thickness of 2 mm and immersed during one week in pentane solvent (C_5_H_12_) to extract resin. The samples were dried again, X‐rayed and the X‐ray films were scanned at 4,000 dpi, then analyzed with WINDENDRO (Windendro 2008e Regent instruments, Canada).

We cross‐dated the tree‐ring time‐series using the *Interdat* software (version 1.1, Becker et al., 1994; Mérian et al., 2011) and pointer years based on the tree‐ring width (RW) measurements provided by WINDENDRO. We considered the rings 1967–2007 (41 successive years). We used the “extreme average method” (Vargas‐Hernandez & Adams, 1991) to separate each annual ring microdensity profile into its earlywood and latewood components. We then obtained the widths (RW), the average densities (RD) of each dated annual ring, specifically the earlywood, latewood width (EW and LW), and the earlywood and latewood average density (EWD, LWD).

We used and compared several methods to correct the time‐series of ring microdensity variables to only keep climatic variability (Rozenberg, Chauvin, et al., 2020). Our objective was to estimate individual RN on the maximum number of trees. However, fitting RN with statistically significant slopes is often a big challenge in PP studies. Here we compared different methods used at the successive steps of the RN construction, for their ability to maximize the number of trees with significant RN. First we tested several detrending methods: we used no correction, negative exponential curve (Cook & Kairiukstis, 1990; Fritts, 1976) and regional curve standardization (RCS; Esper et al. 2003), with residual RCS and ratio RCS (Esper et al., 2003; Rozenberg, Chauvin, et al., 2020). For the negative exponential function we used the dendrochronology library in R, dplR (Bunn, 2008). Since larch budmoth (LBM) can induce major cyclic defoliations with important non‐climatic impact on ring microdensity profiles (Castagneri et al., 2020; Peters et al., 2020; Rozenberg, Pâques, et al., 2020) we also tested the impact of standardizing for this effect. For each individual tree and for all annual rings with a validated *Zeiraphera griseana* attack within a plot, the values of the corresponding ring variables were replaced by the average value of the three previous and the three subsequent annual rings (Büntgen et al., 2009; Rozenberg, Pâques, et al., 2020).

### 2.3 Construction of the reaction norms and estimation of phenotypic plasticity

To fit the reaction norm (RN) between the tree‐ring variables and the climate variables we applied the linear model *Y*_i_ = β1 + β2*X*_i_ + ϵ_ij_ using the lm() function of R (R Core Team, 2018), where *Y*_i_ is the phenotypic variable; *X*_i_ is the climatic variable, β1 is the intercept and β2 is the slope (the PP value), and ϵ_ij_ is the error term (the residuals). The adjusted model R‐square is used to estimate the quality of the relationships. When *p* is under 0.05 we assume that the RN slope is significantly different from zero and can be used as a quantitative estimate of the PP.

The climatic data are daily values of minimum and maximum temperature and precipitation coming from the closest Météo‐France weather station (French national weather service; Rozenberg, Chauvin, et al., 2020). This weather station moved from Briançon (44°53'59.21" N, 6°38'31.24" E, 1,306 m) to Villard‐St‐Pancrace (44°51' 57.5388'' N, 6°36'58.7772'' E, 1,276 m), 4.8 km south of the Briançon weather station, in 2003. Nevertheless, from 2004 to 2010 both weather stations have been operating together. Thus, for this study (1966–2007) we used the Briançon data only. For each plot we adjusted the Briançon climatic time‐series from the elevation effect, as in (Latreille et al., 2017; Rozenberg, Chauvin, et al., 2020) using the on‐site climatic data measured since 2008 at each plot.

We estimated the RN by averaging the daily climatic data for time‐windows of variable duration and position during the calendar year. We compared four methods (M1 to M4). In M1 we fitted the RN with the same annual climatic means for all the trees in the four plots. In M2 we estimated the RN for a time‐window based on the results of a ring formation study (Saderi et al., 2019). It reflects the duration of the ring formation period estimated on a sub‐sample of the same trees, studied during the 2013 growing season (Saderi et al., 2019). In M3 we systematically tested different time‐windows and at each elevation level. We selected one common time‐window for all the trees in each plot: this time‐window is the one giving the highest number of trees with significant RN. Finally in M4, we tested the time‐windows at the tree level: for each tree we selected the time‐window giving the RN with the highest R‐square. We obtained similar results for minimum and maximum temperature. M3 and M4 gave a higher proportion of trees with significant RN than M1 and M2. In contrast we were not able to fit RN with statistically significant slopes (*p* < .05) with precipitation, whatever the method, which was then excluded from the study. Figure S2 in Supporting Information shows the heatmap of the tested flexible time‐windows in M4 for a given tree, for LWD and maximum temperature.

The slopes are used as quantitative estimates of the tree PP. We used a single factor fixed effect ANOVA to test for the differences between the elevational plots.

## 3 RESULTS

### 3.1 Diversity of the RN along the gradient

The main characteristics of the trees in the four elevational plots (table 2) showed that on average the trees were slightly bigger at 1,700 m, taller at 1,700 and 2,000 m, younger at 2,300 m and with a lower wood density at 1,350 m.

**TABLE 2:** Characteristics of the trees and mean values of the ring variables at each elevation plot

Elevation (m)	Number of trees with microdensity profiles	Height (m)	Estimated average age (years)	Circumference at breast height (cm)	Average RW (mm)	Average EWD (g/dm^3^)	Average LWD (g/dm^3^)	Average ring density (g/dm^3^)
2,300	135	16 (±4.83*)	95.7 (± 34.1*)	81 (±0.38*)	0.91 (±0.43*)	0.39 (±0.05*)	0.75 (±0.10*)	0.49 (±0.06*)
2000	124	26 (±6.46*)	134.0 (± 48.8*)	101 (± 0.48*)	0.63 (±0.26*)	0.39 (±0.05*)	0.74 (±0.12*)	0.50 (±0.06*)
1,700	154	27 (±6.22*)	133.2 (± 46.1*)	112 (±0.43*)	0.64 (±0.33*)	0.38 (±0. 05*)	0.77 (±0.12*)	0.50 (±0.07*)
1,350	150	24 (±4.66*)	143.7 (± 15.5*)	107 (±0.33*)	0.65 (±0. 32*)	0.33 (±0.05*)	0.73 (±0.14*)	0.45 (±0.07*)

* Standard deviation.

#### 3.1.1 Test and selection of the methods (standardization and construction of RN)

We observed that the standardization method to adjust from the cambial age effect did not affect the results: the number of trees with significant RN was not strongly modified and the results were the same with the different standardization methods tested. We also found that the larch budmoth standardization did not affect the results since it did not change the general findings (data not shown).

**FIGURE 1:**
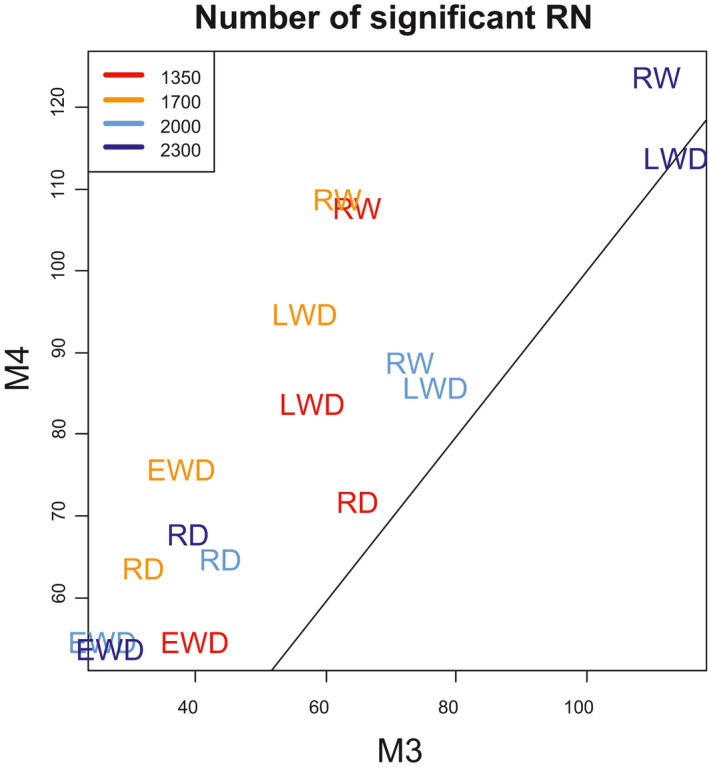
Comparison of the number of trees with significant RN obtained with methods M3 and M4 for each ring variable and at each elevation. The line shows the *y* = *x* line

In contrast, the number of trees with significant RN varied a lot between the four fitting methods M1 to M4. M3 and M4 gave the highest number of trees with significant RN. For most ring variables and at most elevations, the number of trees with significant RN was higher for M4 than for M3, with a difference ranging from six to 46 trees, corresponding to four to 30% of the total number of trees in the plot (Figure 1). The superiority of M4 over M3 was stronger for RW at 1,350 and 1,700 m and for EWD and LWD at 1,700 m (Figure 1). The only exception was LWD at 2,300 m, where there were 115 trees with significant RN with M3 and 114 for M4 (Figure 1).

**FIGURE 2:**
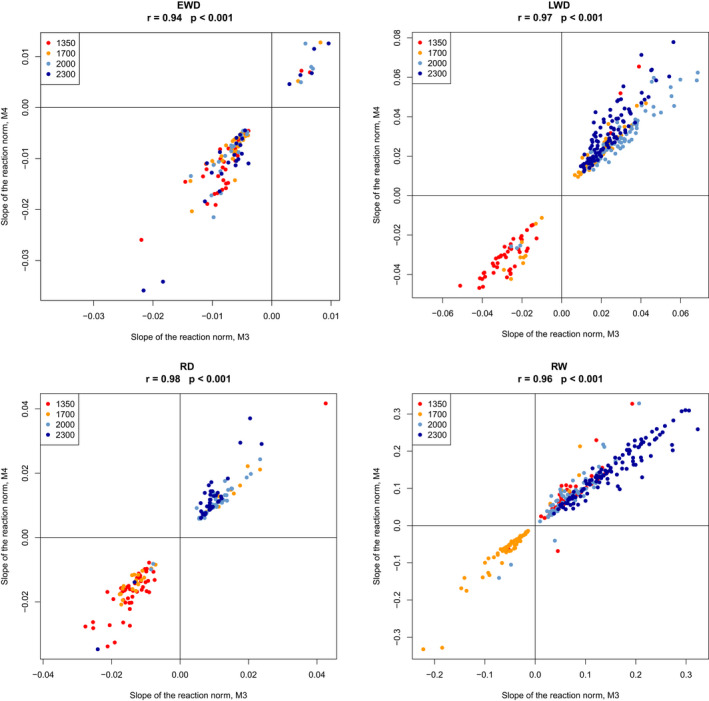
Comparison of the values of the slope of the RN in M3 and M4 for the four ring variables at the four elevation plots

We also compared the value of the slopes of the RN fitted with M3 and M4. We found that they were very strongly correlated, with r ranging from 0.94 to 0.98 according to the ring variable (Figure 2).

Finally, we observed that, when significant, the main trends of the findings were similar between M3 and M4. The higher number of trees with estimations of PP in M4 made that, compared to M3, more results concerning the elevation trends were significant.

#### 3.1.2 Reaction norms (RN)

**FIGURE 3:**
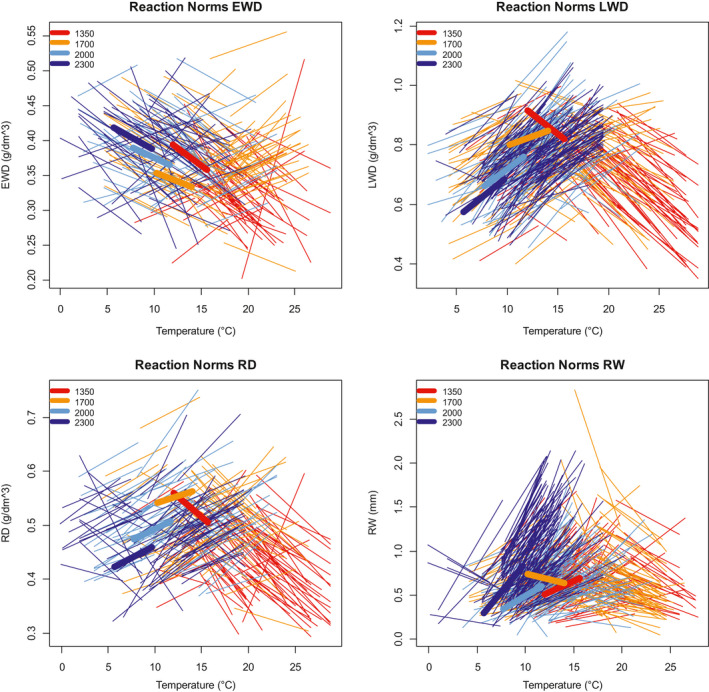
Plot of the individual reactions norms, grouped by ring variable and elevation. Thick lines indicates the average slope for each elevation. All individual slopes are significantly different from zero. Only the average slopes of RW and LWD at 2,000 and 2,300 m a.s.l. and of RD at 2,000 m a.s.l. are significantly different from zero

We succeeded to estimate RN relating ring variables and minimum and maximum temperature with significant slopes for the majority of the trees in the four elevation plots with method M4. The results obtained with minimum and maximum temperature were extremely similar, almost redundant, with a slightly better fitting with maximum temperature for all the ring variables. Thus, we decided to retain maximum temperature. In the rest of the section, we present only the results obtained with maximum temperature and M4. On average, 71% of the trees displayed a RN with a significant slope (*p* < .05). The significant reaction norms that we estimated at the individual tree level were all linear, with a median correlation coefficient with maximum temperature of 0.44 (ranging from 0.31 to 0.99, with the first quartiles at 0.37 and 0.53). The number of individuals with significant slopes was higher for RW and LWD than for EWD and RD (Figure 1). All individual RN with significant slopes grouped by ring variables showed different mixtures of positive and negative slopes (Figures 2 and 3) with a more stable pattern between elevation levels in RW and LWD. If slopes are averaged by elevation, only four site‐specific slopes resulted to be higly significant (*p* < .01), namely RW and LWD at 2,000 and 2,300 m a.s.l. (Figure 3; Figure S3 in Supporting Information) whereby the two at 2,300 were stronger than at 2,000 m. The other site average RN slopes were not significant, except for RD for which we found a significant and low relationship at 2,000 m a.s.l. (r = 0.38, *p* =.014, not shown).

**FIGURE 4:**
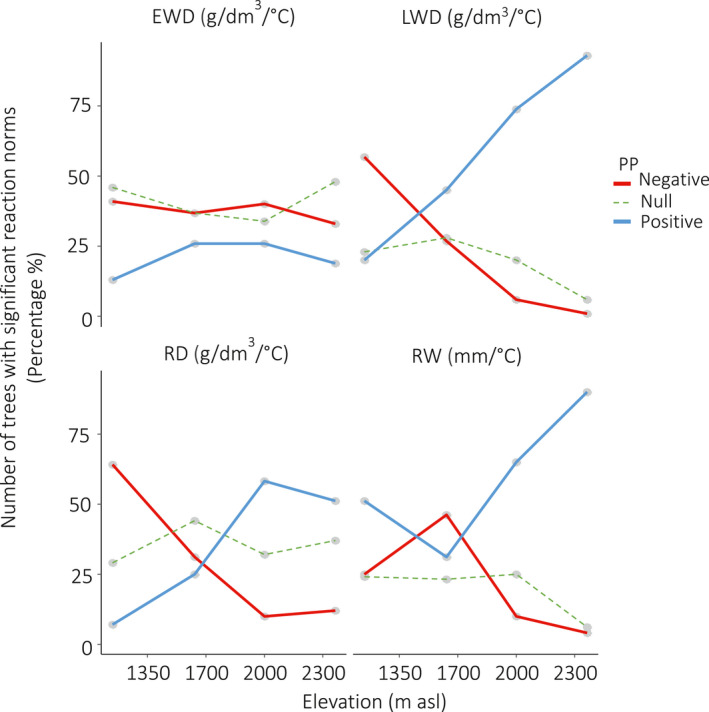
Percentage of trees with significant and non‐significant reaction norms (method M4): trends by variable, elevation and type of PP. Blue line = Positive PP, Red line = Negative PP, Green line = Null (not significant) PP

Figure 4 presents the percentage of trees with significant slopes for each ring variable at each elevational plot and grouped by positive (the slope of the RN is positive), negative (the slope is negative) and null (the slope is not significantly different from zero) PP (the corresponding numbers are in Table S2 in Supporting Information). Depending on elevation, the percentage of significant slopes ranged between 53% and 66% for EWD, 73 and 94% for LWD, 63 and 71% for RD and 75 and 94% for RW (Figure 4). For EWD and RD at all elevations, and for LWD and RW from 1,350 to 2,000 m, the range of percentage of trees with significant slopes varied from 3% to 13%. For LWD and RW, the percentage peaked at 2,300 m, where it reached 94% for both ring variables.

#### 3.1.3 Time‐windows

The individual time‐windows used to estimate the significant RN in M4 are presented in the Supplementary Material (Table S3; Figures S4–S7 in Supplementary Material). These time‐windows were very variable among the ring variables, elevations and trees. The variation of the characteristics of the time‐windows (first day, last day and duration) between the elevations was mostly significant for the first and last days, but not for the duration (Supplementary Material: Figures S5–S7). When significant, these elevational trends were increasing for both the first and last days for positive slopes and most of the time decreasing for both the first and last days for negative slopes. In other words, both the first and last days of the maximizing time‐windows were postponed with increasing elevation for the positive slopes and advanced for the negative slopes. The length of the time‐windows was generally not significantly variable between elevations, except for EWD negative slopes and for RW positive slopes.

#### 3.1.4 Variation of the slope value and of the PP intensity

**FIGURE 5:**
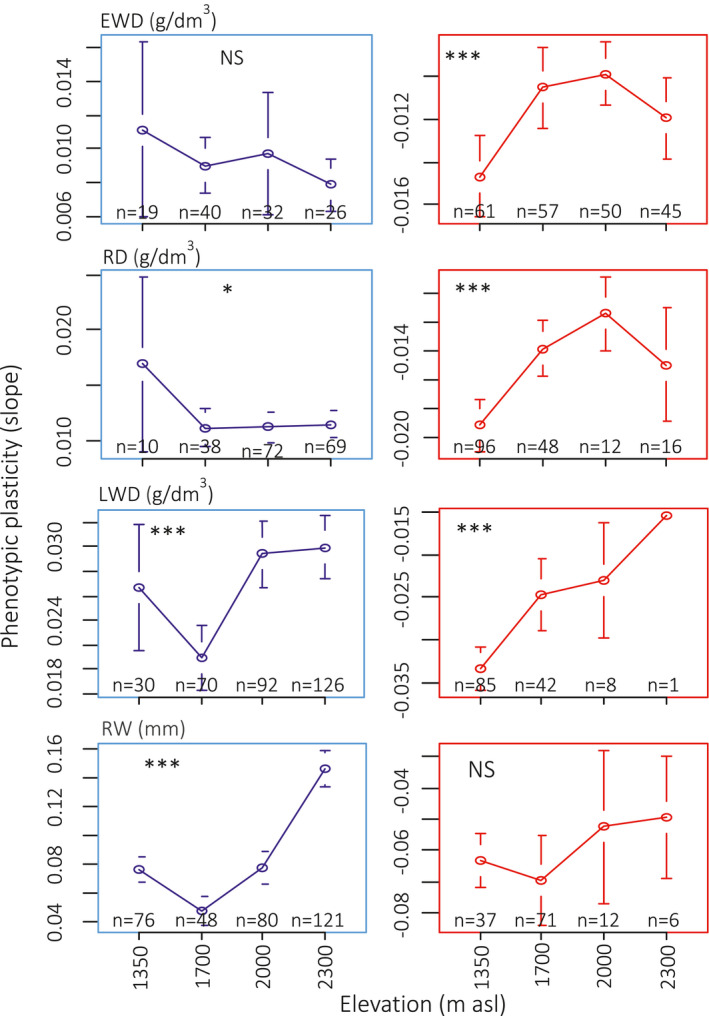
Variation of the PP values between the variables and the elevational levels. Blue line = Positive PP and Red line = Negative PP. Error bars represent standard error of the mean. Signif. codes: NS: *p* ≥ .05, *: *p* < .05, **: *p* < .01, ***: *p* < .001

Inside each group of slopes, positive and negative, there was variation of the value of the slope, that is, variation of the intensity of PP. For most variables and types of PP this intensity of PP is significant between elevational levels (Figure 5). In two cases only (EWD for positive PP and RW for negative PP) the elevational variation was not significant. In one case (RD for positive PP) the variation was significant at the 5% probability level. The corresponding trend was a consequence of steeper slopes at 1,350 m (Table 3). But the corresponding number of trees was only 10, while it was respectively 38, 72 and 69 at the other elevational levels, between which there was no significant variation of PP (Figure 5; Table 3). For the five other cases, the variation of the PP values was significant at the 0.1% probability level. In these cases, the highest PP values (positive and negative) were always found at 2,000 or 2,300 m a.s.l., while the lowest were always found at 1,350 or 1,700 m a.s.l. However, in some cases, these minimum and maximum PP values were estimated with a low or very low number of trees: for RD, the mean value of the negative PP at 2,000 m a.s.l. was estimated with 12 trees. Consequently, there was no significant difference between the three higher elevations (1,700, 2,000 and 2,300 m a.s.l., Table 3). For LWD at 2,000 and 2,300 m a.s.l., the mean value of the negative PP was estimated with respectively eight and one trees only. Here again, there was no significant difference between the three higher elevations. The significant elevational variation of the PP value was better described for LWD (positive and negative PP), RW (positive PP) and for EWD (negative PP) (Figure 4). The details about the significance of the variation between pairs of elevational levels in Table 3 show that for LWD positive PP, the value at 1,700 m was strongly and significantly lower than that of the PP at the higher elevational levels. For RW positive PP, the elevational trend was highly significant between each pair of successive elevational levels. Finally, for EWD, only the value of the negative PP at 1,350 m asl was significantly different from that at the three higher elevations (Table 3).

**TABLE 3:** Significance of the two‐by‐two differences between the elevational levels, for positive and negative slopes of the RN. PP – Pos = Positive PP, PP – Neg = Negative PP, Blue = Positive PP (Pos) and Red = Negative PP (Neg). Signif. codes: NS: *p* ≥ .05, *: *p* < .05, **: *p* < .01, ***: *p* < .001

Variable	Elevation	PP ‐ Pos	PP ‐ Neg	Variable	Elevation	PP ‐ Pos	PP ‐ Neg
EWD	1,350–1,700	NS	**	RD	1,350–1,700	*	***
	1,350–2,000	NS	***		1,350–2,000	*	**
	1,350–2,300	NS	*		1,350–2,300	*	NS
	1,700–2,000	NS	NS		1,700–2,000	NS	NS
	1,700–2,300	NS	NS		1,700–2,300	NS	NS
	2,000–2,300	NS	NS		2,000–2,300	NS	NS
LWD	1,350–1,700	*	***	RW	1,350–1,700	***	NS
	1,350–2,000	NS	*		1,350–2,000	NS	NS
	1,350–2,300	NS	NS		1,350–2,300	***	NS
	1,700–2,000	***	NS		1,700–2,000	***	NS
	1,700–2,300	***	NS		1,700–2,300	***	NS
	2,000–2,300	NS	NS		2,000–2,300	***	*

## 4 DISCUSSION

In this study, we relied on 41 years tree‐ring long time‐series from 821 trees to successfully estimate individual phenotypic plasticity (PP) of *Larix decidua* micro‐density ring variables in response to changes in temperature. The study was conducted for four different ring variables measured on four large groups of trees distributed along an elevational gradient characterised by a 6°C amplitude between the lowest (1,350 m) and highest (2,300 m) plots. Based on inter‐annual temperature variations, we succeded to estimate reactions norms (RN with significant slopes) to average maximum daily temperature for 71% of the 555 adult trees.

We found individuals with slopes of reaction norms in opposite directions for different plasticities, that is, different associations of phenotypic and environmental variables. This is common and reflects the specific physiological process associated with each phenotypic variable (Pélabon et al., 2013). For each of the four ring variables studied, we found a broad range of individual tree plasticities within a natural population: for a given trait, they range from positive to negative slope values. This population includes as well a more or less large proportion of individuals (from 6% to 41%) with no PP related to the climatic variable studied (null plasticity).

There are other examples of slopes of reaction norms in opposite directions for a given PP in animals (fishes and insects) and in plants, but we found none in trees. The variation between positive and negative slopes was sometimes associated with age (Smoliński et al., 2020), more often with quality of environment (Diamond & Kingsolver, 2012; Tammaru & Teder, 2012; Teder et al., 2014), and in the case of field gentian (Gentianella campestris), with population variation (Juenger et al., 2000). In our case, the variation between positive and negative slopes slopes of the RN is related with elevation for three of the four ring variables. Across all variables, the slopes have a tendency to be more negative at the low elevation and more positive at the top. Furthermore, the individuals with a negative slope at the low elevation plots (1,350 and 1,700 m) tend to have a more negative slope than the individuals from the high elevation plots (2,000 and 2,300 m). The more positive slopes at the higher elevation plots for the trees with a positive slope were found mainly for RW and LWD.

Our results mean that a temperature increase is indeed associated to opposite reactions between trees within a population: the temperature increase corresponds, according to individual trees, to either an increase or decrease of ring width, ring density, earlywood density and latewood density (Figure 3). The very strong relationship between the slope values estimated with fixed time‐windows at each elevation and with variable time‐windows at each elevation (Figure 2) suggests that the reason behind this is not the selection of different time‐windows for the different trees. This is confirmed by the fact that the other results of the analysis using fixed time windows and flexible time‐windows at each elevation level were very similar (same differences between the ring traits and same elevational variation), at the cost of a much lower number of trees with significant estimation of PP (results not shown).

### 4.1 Strength and weaknesses of the flexible time‐windows

As stated immediately above, we carefully compared the results of the analysis conducted with fixed and flexible time windows. We observed that the number of trees with significant reaction norms was lower with the fixed time windows method. The flexible time window method better took into account the differential behaviour of the individuals towards temperature. If at species or even population levels, mean annual (or growing season) temperature is convenient and satisfactory to explain overall radial tree growth, it may not be the case anymore when the target is the individual response. One consequence was that, especially for earlywood density, the statistical power of between‐elevation‐level comparison decreased for the fixed‐window method in comparison with the flexible‐window method: some of the differences between the elevation levels were not statistically significant. We also screened the strength of the relationships between the ring variables and temperature for each individual using correlation heat‐maps for the flexible time‐windows (example tree 41 for LWD, Figure S2 in Supplementary Material). The results showed that for most trees, the range of the first and last days of the time‐windows displaying significant correlation was usually large and covered a wide period. For example (Figure S2 in Supplementary Material), a change in the correlation coefficient value from 5% to 10% is observed within a 2 to 3 months time‐window change. It means that in the tested range, the choice of the time‐window did not affect much the value of the correlation coefficient, because it did not affect much the interannual variation of the average temperature of the time‐window. Conversely, the temperature‐sensitive periods were rather variable between the ring variables and the elevational plots (Figures S4–S7, Supplementary Material). As expected, days that maximize the relationship with ring growth and density parameters are delayed at higher elevations compared to lower elevations. If we assume that there is a close relationship between these time‐windows and the phenology of ring formation, we would expect for example earlier periods for earlywood than for latewood (Rossi et al., 2013; Saderi et al., 2019) and shorter periods for the high elevation plots (He et al., 2012; King et al., 2013). However the variation observed among plots and traits is not fully consistent with what could be expected from a biological point of view. The time‐windows we defined are not supposed to precisely determine starting and ending days of growth processes but are rather climatic periods maximizing statistical relationships without any attempt to physiologically interpret them. These annual periods are convenient tools to maximize the number of trees with significant estimations of the RN and to increase the statistical power of the experiment, but should not be strictly interpreted from a biological point of view.

### 4.2 Variation of phenotypic plasticity (PP) along the elevation gradient

We used the significant (positive or negative) slopes of the RN as quantitative estimates of PP. For the trees with non‐significant RN, we cannot conclude if the non‐significant estimation of the slope corresponds to a flat reaction norm (Fusco & Minelli, 2010), or null PP, or to a failure to estimate the PP (the individual is plastic but we were not able to estimate its PP with the available data). For convenience, we call trees with “null PP” the trees for which the estimation of the slope was not statistically significant (not significantly different from zero). For an adaptive trait, the distinction between positive and negative slopes could correspond to a major (genetic?) divergence between responses. For latewood density, and to a lesser extent, for ring width and ring density, the change from negative to positive slopes is closely associated with elevational variation. The proportion of trees with a positive PP tends to increase from the bottom to the top of the gradient, with obviously the opposite trend for the negative PP. This tendency is especially strong for LWD (latewood density): in the warmer conditions at the bottom of the gradient, the temperature increase is associated to a latewood density decrease for most of the trees, while in the colder conditions at the top of the gradient, a temperature increase is associated to a latewood density increase for nearly all the trees. This trend is less marked for RD (mean ring density), which logically shows a pattern intermediate between that of earlywood and latewood density. The results in Figures 3, 4, 5 provide a global picture of PP variation along this elevational gradient: the PP tends to be more negative in the warmer conditions at low elevation, while it is mostly positive in the colder conditions at high elevation. There is some variation around this trend, since trees with opposite tendencies are found all along the gradient, although in very variable proportions. The heterogeneity of the mixture is greater at the lower, warmer elevations (1,350 and 1,700 m asl) and smallest in colder environments, at 2,000 m and above. The 1,700 m plot appears as a kind of hotspot of PP diversity.

In the case of earlywood density (EWD), the proportion of trees with positive and negative PP does not change much with elevation, with a greater proportion of trees with a negative PP at all elevations: contrary to the other ring variables, most part of the contrast between positive and negative PP is within plots. The mixture of trees with such contrasting PP values at the same elevational plot, comprising negative and positive PP, explains well the non‐significant average plastic response found for RD and EWD at all elevations, and for RW and LWD at 1,350 and 1,700 m asl (Figure 3; Figure S3 in Supporting Information). A direct consequence is that plastic response, as well as other plasticity index used in dendroecology studies (de Luis et al., 2013; Martínez‐Vilalta, 2018; Matisons et al., 2019; Paiva et al., 2008; Sánchez‐Salguero et al., 2018) correspond to particular cases of PP at the population or species level. It certainly hides sometimes large individual variation of PP. In our study, strong or moderate inter‐individual variation of PP for RD and EWD at all elevations and for RW and LWD at 1,350 and 1,700 m a.s.l. lowers the intensity of the corresponding average plastic response, making it non significant. Information at this level is however key to study the PP potential of evolutionary adaptation, which relies on phenotypic and genetic variation, and on heritability.

The linear warming trend observed at Briançon explains about half of inter‐annual temperature variation between 1967 and 2007 (Figure S1 in Supporting Information). In another study, a similar trend was found for the 1967–2016 period at the same location (Rozenberg, Chauvin, et al., 2020). If we assume that this linear temperature increase is a manifestation of the global warming, then about half of the PP is an individual response to this global warming. According to (Rozenberg, Chauvin, et al., 2020), this global warming effect is stronger for tree response at low elevation than at high elevation: global warming reveals the potential of plasticity of larch. Whether such response can be considered as an adaptation mechanism relies on the adaptive value, or fitness, of PP. To what extent the fitness of PP is related to the fitness of the phenotypic trait (Nicotra & Davidson, 2010; Via et al., 1995)? In our case, direct relationship with fitness is not available, thus the putative adaptive value of PP can only be inferred from the possible functional role of the associated phenotypic trait. This role is mainly related to the three wood functions, mechanical support, reserves and sap conduction which are related to the four ring variables for which we estimated PP. As discussed above, the modification of wood anatomical and hydraulic properties associated to the observed PP is a promising research line that requires further investigations.

**FIGURE 6:**
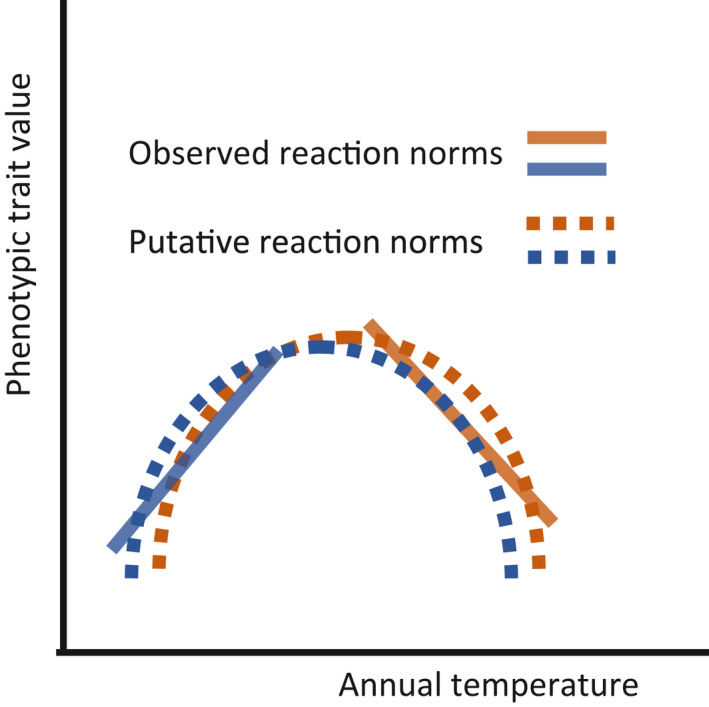
The linear reaction norms observed at the bottom (orange with a negative slope) and at the top (blue with a positive slope) of the elevation gradient could be segments of unique nonlinear reaction norms. Whether both putative reaction norms are similar (as suggested by the figure) or different is unknown

Within plots and across the gradient, the existence of a broad phenotypic variability for individual tree reaction norms (in sign and intensity) is a favorable indication and one of the conditions for populations to evolve through natural selection and hopefully to adapt to new climatic conditions. The jump from negative to positive PP could correspond to dramatically opposed individual adaptive strategies. It could correspond to different segments of nonlinear reaction norms. Obviously, and as observable for example in Figure 3, the range of temperature variation at each elevation is shorter than the complete range of interannual temperature variation along the whole gradient. What would be the shape of a reaction norm of a tree facing this complete range of interannual temperature variation? The opposite directions of the slopes of the low and high elevation trees in their respective temperature conditions suggest that a tree facing the full temperature range would respond with a nonlinear reaction norm, maybe similar to the bell‐shaped one shown in Figure 6. This would be consistant with the fact that reaction norms of most plant phenotypic traits observed accross a wide temperature range are expected to be nonlinear (Arnold et al., 2019). A way of testing this hypothesis could be to plant reciprocal transplant experiments: if the assumption is correct, vegetative copies of low and high elevation trees transplanted at the other extremity of the gradient would respond with an inverted slope.

**FIGURE 7:**
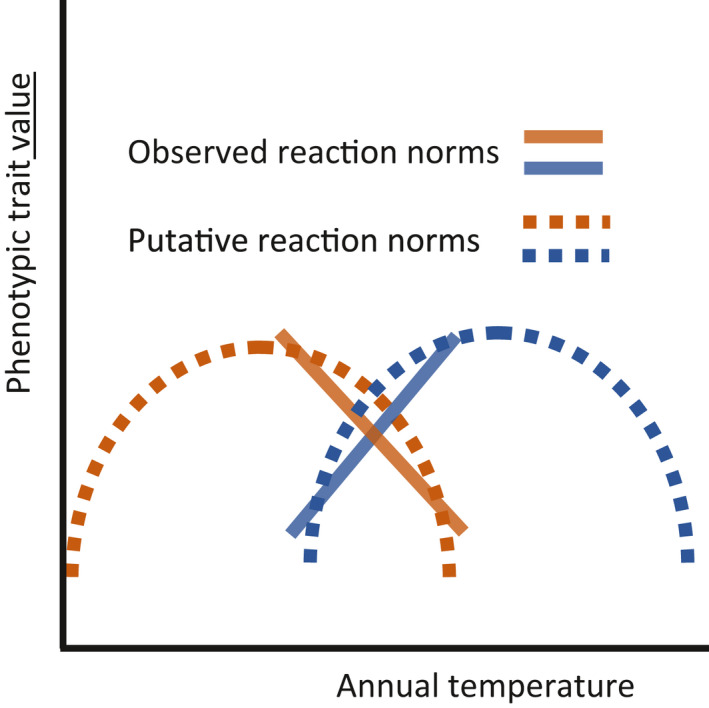
Two trees with opposite slopes of reaction norms at the same elevation level may have in reality reaction norms with similar nonlinear shape but shifted along the temperature axis

For some ring variables and/or at some elevation, there is a mixture of trees with positive and negative slopes. Such opposite PP could correspond to differences between trees for the position of the complete nonlinear reaction norm along the temperature axis, as shown in Figure 7.

This variation could reflect both environmental and genetic differences between the trees. The proportion of between‐tree environmental and genetic variation could be very different for trees sharing the same plot or being largely separated along the gradient. However, test of these hypotheses requires access to genetic information, which necessitates other approaches such as genetic trials. The experimental trial in this study does not encompass a common garden experiment and thus does not permit the accurate separation of the environmental and genetic components of PP variation. A previous study of genetic diversity along the same elevational gradient (Nardin et al., 2015) showed a genetic structure only slightly affected by climatic variation, human activities or historical events. Yet, a small but significant inter‐plot genetic variation indicates the existence of variable genetic dynamics, which could be an indication of local adaptation (Nardin et al., 2015). This suggests that the strong gene flow between all elevations levels redistributes more or less the same large genetic diversity everywhere at each new generation. This large genetic diversity could be related with the large PP variation observed along the gradient and especially at mid‐gradient. Indeed, large phenotypic variation is often associated to large genetic variation (Roff, 1995). In addition, the more constraining climatic conditions at the gradient extremities could locally select adapted trees and reduce phenotypic and genetic variation, including for PP. But these facts are not enough to provide evidence for genetic variation, local adaptation and a potential for evolutionary adaptation for PP in this gradient. Studies based on genetic trials, reciprocal transplant experiments and non‐neutral genetic markers would deliver additional relevant information.

### 4.3 Strength of the individual reaction norm approach

A PP variable obtained from the reaction norm can be seen as a complex *dynamic* phenotypic trait. This new dynamic trait encompasses the variation of the original phenotypic variable within a given range of environmental variation. Earlier studies using tree‐rings have often assessed average plasticity by quantifying the environmental response at the population level using main site tree‐ring chronology (Fonti et al., 2010; de Luis et al., 2013) and the RDPI (Valladares et al., 2006) of xylem traits (Scholz et al., 2014). In other cases, inter‐ring average plasticity was discussed based on indirect estimations and tolerance analysis (Matisons et al., 2019) or climate‐growth plastic responses (Arzac et al., 2018; Caminero et al., 2018; Sánchez‐Salguero et al., 2018). In the few studies investigating inter‐ring PP with individual reaction norms, the results do not contain information about the proportion of trees with significant estimations (Fallour‐Rubio et al., 2009; Marchal et al., 2019). Information about individual reaction norms becomes particularly relevant to evaluate the fate of populations facing strong selection pressure such as imposed by climate changes and how their diversity might prepare them to adapt.

The retrospective construction of individual RN using inter‐annual ring variation is a very promising tool for the study of PP. Intra‐individual variation in RN, that is, reiterated expression of RN during an individual's lifetime (Araya‐Ajoy et al., 2015), could also be based on tree‐ring analysis, taking advantage of the strong intra‐ring (from earlywood to latewood) variation for basic wood properties (anatomy, micro‐density). First theoric and practical attempts (Fonti & Jansen, 2012; Martinez‐Meier et al., 2009; Sánchez‐Vargas et al., 2007) support the feasibility of the method. Random regression mixed model is another promising framework for studying plant PP to global warming and its associated environmental and genetic determinism in an accurate and integrated way (Arnold et al., 2019; Fallour‐Rubio et al., 2009; Marchal et al., 2019), as long as the number of estimated individual RN is high enough.

Whereas this study shows that PP tends to be greater in the colder conditions at the top of the gradient, and more variable in the most favourable conditions at mid gradient, there are also very strong differences between ring variables: it does not seem possible to consider PP as a single trait. PP should be systematically referred to mentioning the corresponding phenotypic trait‐environmental variable association. Estimating reaction norms at the individual tree level provides information about the inter individual variation of PP, which is not available when it is studied as the average plastic response of a group (species, population) of trees. This information is especially relevant in evolutionary studies when the adaptation potential of the population submitted to new selection pressure such as climate change is heavily dependent of the available genetic diversity of its individual tree components.

## CONFLICT OF INTEREST

The authors declare no conflict of interest.

[Correction added on 18 June 2021, after first online publication: Conflict of Interest statement added to provide full transparency.]

## AUTHORSHIP

Margarita Escobar‐Sandoval contributed to methodology; software; formal analysis; investigation; visualization; and writing ‐ original draft. Luc Pâques contributed to conceptualization; investigation; writing ‐ review & editing; and supervision. Patrick Fonti contributed to investigation; writing ‐ review & editing; and supervision. Alejandro Martinez‐Meier contributed to investigation; writing ‐ review and editing. Philippe Rozenberg contributed to conceptualization; methodology; software; formal analysis; writing ‐ review & editing; visualization; supervision; project administration; and funding acquisition.

## Supporting information

Supplementary Material

## ENDNOTE

^1^
“Significant” refers to the statistical meaning of the word: we call “significant RN” a RN for which the slope of the associated linear relationship is significantly different from zero.
