# Supplementary material for: Phenotypic plasticity of European larch radial growth and wood density along a‐1,000 m elevational gradient
Source: Plant Environ Interact. 2021 Feb 20;2(2):45–60. doi: 10.1002/pei3.10040 (PMC10168074; doi:10.1002/pei3.10040)
Supplement: Supplementary file 1 — Supplementary Material [file PEI3-2-45-s001.docx]

**Supplementary material**

Table S1: Geographical, structural and ecological features of the elevational plots.

| \|  \| 2300 m \| 2000 m \| 1700 m \| 1350 m \| amplitude \| CV \| \| --- \| --- \| --- \| --- \| --- \| --- \| --- \| \| Elevation range (m) \| 2357-2299 \| 2023-1988 \| 1683-1640 \| 1373-1341 \| - \| - \| \|  \|  \|  \|  \|  \|  \|  \| \| *Climatic variables for the period 1967-2007:* \|  \|  \|  \|  \|  \|  \| \| Mean annual temperature \| 2.92 \| 4.46 \| 6.32 \| 7.66 \| 4.74 \| 0.39 \| \| Mean number of frost days per year \| 208 \| 192 \| 170 \| 154 \| 55 \| 0.13 \| \| Mean temperature of the warmest month (July) \| 10.95 \| 12.95 \| 15.37 \| 17.09 \| 6.14 \| 0.19 \| \| Mean temperature of the coldest month (January) \| -3.74 \| -2.72 \| -1.49 \| -0.61 \| 3.13 \| 0.64 \| \| Temperature difference between the warmest (1994) and coldest year (1984) \| 2.94 \| 2.83 \| 2.71 \| 2.62 \| - \| - \| \| Inter-annual difference between the mean temperature of the warmest and coldest January month \| 8.39 \| 8.32 \| 8.24 \| 8.19 \| - \| - \| \| Inter-annual difference between the mean temperature of the warmest and coldest July month \| 6.19 \| 5.72 \| 5.25 \| 5.26 \| - \| - \| \| Mean difference between July and January mean temperature \| 14.69 \| 15.67 \| 16.85 \| 17.70 \| - \| - \| \| Mean difference between July and April mean temperature \| 11.28 \| 11.36 \| 11.46 \| 11.54 \| - \| - \| \|  \|  \|  \|  \|  \|  \|  \| \| *Other environmental factors:* \|  \|  \|  \|  \|  \|  \| \| Mean solar radiation (J/cm²/days) \| 981 \| 927 \| 1270 \| 1324 \| 397 \| 0.18 \| \| Soil water availability (mm) \| 38.7 \| 53.2 \| 66.7 \| 46.5 \| 28 \| 0.23 \| \| Soil fertility index (no unit) \| 236.53 \| 522.51 \| 539.11 \| 147.77 \| 391 \| 0.55 \| \| Soil type \| Calisol \| Eutric Brunisol / Calisol \| Colluviosol \| Regosol / Colluviosol \|  \|  \| \| *Characteristics of trees at each plot:* \|  \|  \|  \|  \|  \|  \| \| Total number of trees investigated \| 198 \| 217 \| 206 \| 200 \| - \| - \| \| Number of microdensity profiles obtained \| 135 \| 124 \| 154 \| 142 \| - \| - \| \| Plot density (nb trees / ha) \| 365 \| 288 \| 354 \| 230 \| 135 \| 0.20 \| \| Area of the plot (square meters) \| 5429 \| 7540 \| 5815 \| 8704 \| 3275 \| 0.22 \| \| Mean age of the trees (years) \| 99.6 (36.4) \| 154.8 (42.7) \| 148.9 (38.7) \| 144.8 (14.0) \| 45 \| 0.18 \| \| Mean diameter (cm) \| 79.3 (27.4) \| 98.6 (32.8) \| 105.1 (34.4) \| 107.0 (25.4) \| 28 \| 0.13 \| \| Mean dominant height (m) \| 16.21 (2.20) \| 25.32 (2.76) \| 26.71 (2.94) \| 23.79 (2.64) \| 7.58 \| 0.20 \| \| Competition index (no unit) \| 120.3 (69.9) \| 130.3 (76.0) \| 147.0 (70.2) \| 120.6 (58.3) \| 28 \| 0.10 \| |
| --- | --- | --- | --- | --- | --- | --- | --- | --- | --- | --- | --- | --- | --- | --- | --- | --- | --- | --- | --- | --- | --- | --- | --- | --- | --- | --- | --- | --- | --- | --- | --- | --- | --- | --- | --- | --- | --- | --- | --- | --- | --- | --- | --- | --- | --- | --- | --- | --- | --- | --- | --- | --- | --- | --- | --- | --- | --- | --- | --- | --- | --- | --- | --- | --- | --- | --- | --- | --- | --- | --- | --- | --- | --- | --- | --- | --- | --- | --- | --- | --- | --- | --- | --- | --- | --- | --- | --- | --- | --- | --- | --- | --- | --- | --- | --- | --- | --- | --- | --- | --- | --- | --- | --- | --- | --- | --- | --- | --- | --- | --- | --- | --- | --- | --- | --- | --- | --- | --- | --- | --- | --- | --- | --- | --- | --- | --- | --- | --- | --- | --- | --- | --- | --- | --- | --- | --- | --- | --- | --- | --- | --- | --- | --- | --- | --- | --- | --- | --- | --- | --- | --- | --- | --- | --- | --- | --- | --- | --- | --- | --- | --- | --- | --- | --- | --- | --- | --- | --- | --- | --- | --- | --- | --- | --- | --- | --- | --- | --- | --- | --- | --- | --- | --- | --- | --- | --- | --- | --- | --- | --- | --- | --- | --- | --- | --- | --- |

Table S2. Number of individual reactions norms (RN) with significant slopes grouped by variable and elevation (M4).

| Ring variable | Elevation | Total RN | Nr of RN with positive slopes | Nr of RN with negative slopes | % positive slopes | % negative slopes |
| --- | --- | --- | --- | --- | --- | --- |
| EWD | 1350 | 150 | 19 | 61 | 13 | 41 |
|  | 1700 | 154 | 40 | 57 | 26 | 37 |
|  | 2000 | 124 | 32 | 50 | 26 | 40 |
|  | 2300 | 135 | 26 | 45 | 19 | 33 |
| LWD | 1350 | 150 | 30 | 85 | 20 | 57 |
|  | 1700 | 154 | 70 | 42 | 45 | 27 |
|  | 2000 | 124 | 92 | 8 | 74 | 6 |
|  | 2300 | 135 | 126 | 1 | 93 | 1 |
| RD | 1350 | 150 | 10 | 96 | 7 | 64 |
|  | 1700 | 154 | 38 | 48 | 25 | 31 |
|  | 2000 | 124 | 72 | 12 | 58 | 10 |
|  | 2300 | 135 | 69 | 16 | 51 | 12 |
| RW | 1350 | 150 | 76 | 37 | 51 | 25 |
|  | 1700 | 154 | 48 | 71 | 31 | 46 |
|  | 2000 | 124 | 80 | 12 | 65 | 10 |
|  | 2300 | 135 | 121 | 6 | 90 | 4 |

Table S3. Signification of the two-by-two difference between pairs of elevation levels for the first and last day and the length of the time windows that maximizes the significance of the reaction norms. Time-windows for trees with positive and negative phenotypic plasticity.

| Variable | Elevation | First day | Last day | Length | First day | Last day | Length |
| --- | --- | --- | --- | --- | --- | --- | --- |
| EWD | 1350 -1700 | * | NS | NS | NS | *** | *** |
|  | 1350 -2000 | NS | NS | NS | NS | ** | * |
|  | 1350 -2300 | NS | NS | NS | NS | NS | NS |
|  | 1700 -2000 | NS | NS | NS | NS | NS | NS |
|  | 1700 -2300 | NS | NS | NS | NS | ** | ** |
|  | 2000 - 2300 | NS | NS | NS | NS | NS | NS |
| LWD | 1350 -1700 | NS | NS | NS | ** | ** | NS |
|  | 1350 -2000 | NS | NS | NS | NS | NS | NS |
|  | 1350 -2300 | *** | *** | NS | ** | NS | NS |
|  | 1700 -2000 | ** | *** | NS | NS | NS | NS |
|  | 1700 -2300 | *** | *** | NS | NS | NS | NS |
|  | 2000 - 2300 | *** | *** | NS | NS | NS | NS |
| MRD | 1350 -1700 | NS | NS | NS | NS | NS | NS |
|  | 1350 -2000 | NS | NS | NS | ** | NS | NS |
|  | 1350 -2300 | NS | ** | NS | *** | *** | NS |
|  | 1700 -2000 | NS | NS | NS | NS | NS | NS |
|  | 1700 -2300 | ** | NS | * | *** | *** | NS |
|  | 2000 - 2300 | *** | * | NS | NS | ** | NS |
| RW | 1350 -1700 | NS | * | NS | NS | NS | NS |
|  | 1350 -2000 | *** | NS | NS | *** | NS | NS |
|  | 1350 -2300 | *** | *** | *** | *** | *** | NS |
|  | 1700 -2000 | *** | *** | NS | *** | NS | NS |
|  | 1700 -2300 | *** | *** | *** | *** | *** | NS |
|  | 2000 - 2300 | * | *** | *** | * | *** | NS |
| PP – Pos=Positive PP, PP – Neg=Negative PP,  Blue =positive PP and Red=negative PP.  Signif. codes: NS: p >= 0.05, *: p < 0.05, **: p < 0.01, ***: p < 0.001 | | | | | | | |


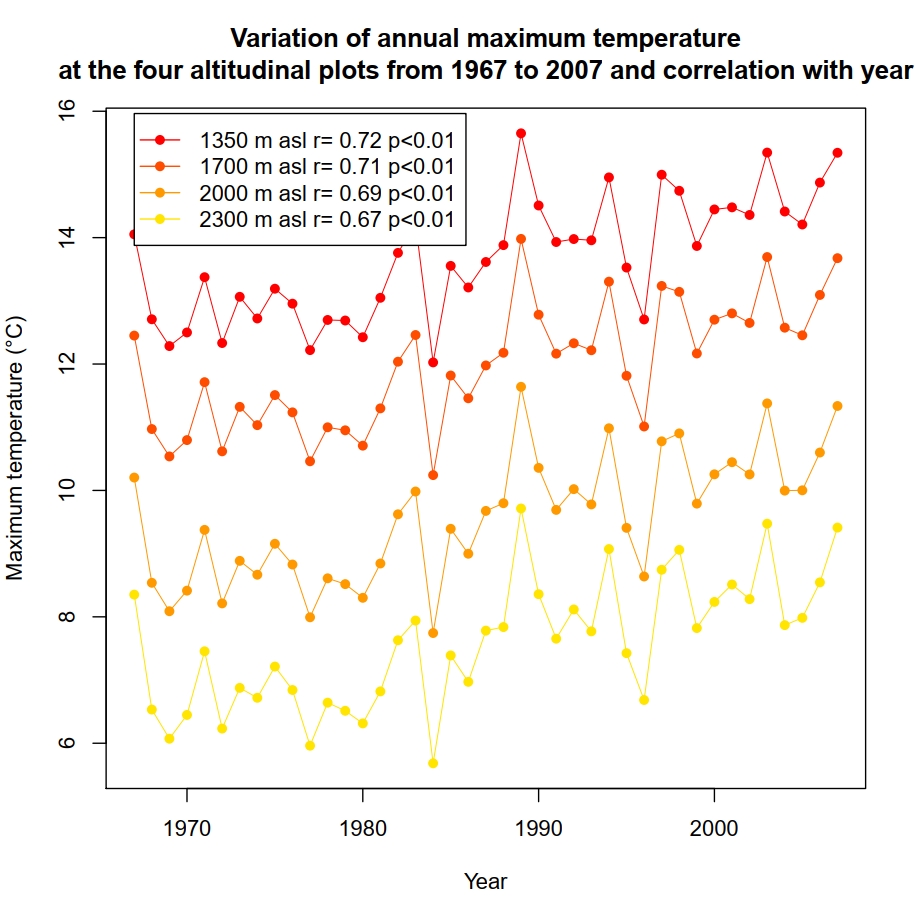


Figure S1. Variation of the annual maximum temperature with time at the four elevational plots, with the corresponding Pearson coefficient of correlation with time (r) and the associated probability (p).


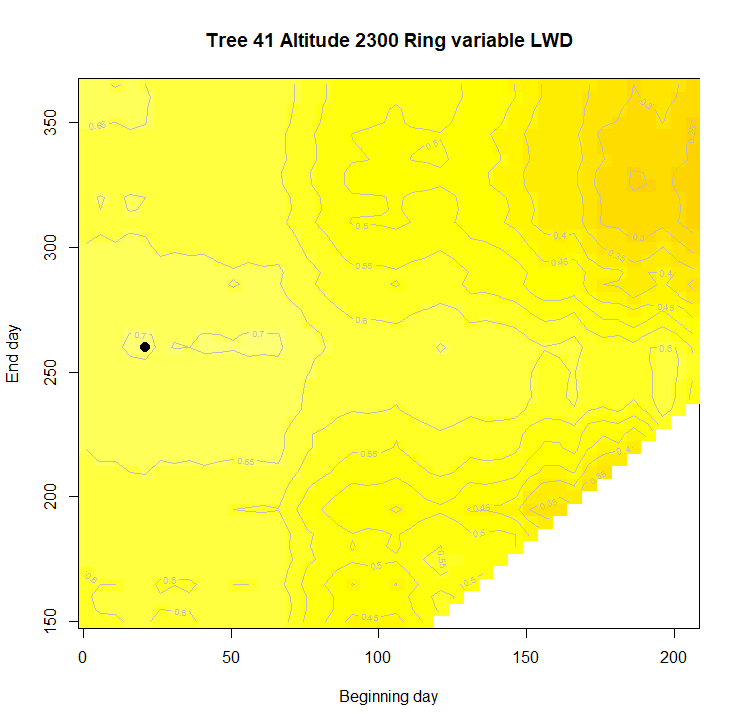


Figure S2. Heat maps and level curves of the distribution of the Pearson correlation coefficients of the correlations between LWD (latewood density) and mean maximum temperature of the time windows defined by the first (beginning) and last (end) days of these periods. This coefficient of correlation measures the quality of the estimation of the PP of the tree 41, located at the 2300 m asl elevational plot.

Figure S3: scatterplot of the relationships corresponding to the four significant average plot reaction norms. RW and LWD with mean annual maximum temperature at the 2000 and 2300 m asl elevational plots, with r the Spearman coefficient of correlation and p the corresponding probability.

Figure S4. Time-windows used to fit the significant reaction norms obtained with the method 4. The x axis is the first and last day of the time-window. The y axis is for the individual trees grouped by elevational plot. The color scheme is for the slope classes.

1700

2300

220

240

260

280

n=19

n=40

n=32

n=26

180

220

260

n=30

n=70

n=92

n=126

160

200

240

n=10

n=38

n=72

n=69

200

250

300

1350

2000

n=76

n=48

n=80

n=121

NS

***

*

***

100

120

140

160

n=19

n=40

n=32

n=26

60

80

120

n=30

n=70

n=92

n=126

60

80

100

140

n=10

n=38

n=72

n=69

50

70

90

110

Altitude (m asl)

First and last day of the period (day of the year).

1350

2000

n=76

n=48

n=80

n=121

LWD (g/dm3/°C)

EWD (g/dm3/°C)

MRD (g/dm3/°C)

RW (mm/°C)

1700

2300

NS

***

***

***

First day

Last day

Figure S5. Variation with elevation of the first and last day of the time-windows that maximize the significance of the reaction norms (day of the year) for positive phenotypic plasticity. Blue =Positive PP. Error bars represent standard error of the mean. Signif. codes: NS: p >= 0.05, *: p < 0.05, **: p < 0.01, ***: p < 0.001

1700

2300

180

220

260

n=61

n=57

n=50

n=45

180

220

260

300

n=85

n=42

n=8

n=1

160

200

240

280

n=96

n=48

n=12

n=16

150

200

250

300

1350

2000

n=37

n=71

n=12

EWD (g/dm3/°C)

LWD (g/dm3/°C)

MRD (g/dm3/°C)

RW (mm/°C)

n=6

First day and last day of the period

Elevation (m asl)

***

*

***

***

80

90

100

110

120

n=61

n=57

n=50

n=45

60

100

140

180

n=85

n=42

n=8

n=1

80

120

160

n=96

n=48

n=12

n=16

0

50

100

150

1350

2000

n=37

n=71

n=12

n=6

1700

2300

NS

**

***

***

First day

Last day

Figure S6. Variation with elevation of the first and last day of the time-windows that maximize the significance of the reaction norms (day of the year) for negative phenotypic plasticity. Red=Negative PP.Error bars represent standard error of the mean. Signif. codes: NS: p >= 0.05, *: p < 0.05, **: p < 0.01, ***: p < 0.001

| Length of the period (days)  Elevation (m asl)  EWD (g/dm3/°C)  90  110  130  n=19  n=40  n=32  n=26  100  140  n=61  n=57  n=50  n=45  120  140  160  n=30  n=70  n=92  n=126  80  120  160  n=85  n=42  n=8  n=1  80  100  120  140  160  n=10  n=38  n=72  n=69  80  120  160  n=96  n=48  n=12  n=16  100  140  180  1350  2300  n=76  n=48  n=80  n=121  100  140  180  1350  2000  n=37  n=71  n=12  n=6  LWD (g/dm3/°C)  MRD (g/dm3/°C)  RW (mm/°C)  1700  2000  1700  2300  NS  ***  NS  NS  NS  NS  ***  NS |
| --- |

Figure S7. Variation with elevation of the length of the time-windows that maximize the significance of the reaction norms, for positive and negative phenotypic plasticity. Blue=Positive PP and Red=Negative PP. Error bars represent standard error of the mean. Signif. codes: NS: p >= 0.05, *: p < 0.05, **: p < 0.01, ***: p < 0.001
